# miRNA and mRNA Profiles in Ventral Tegmental Area From Juvenile Mice With Companion Communication of Improving CUMS-Induced Depression-Like Behaviors

**DOI:** 10.3389/fpsyt.2021.634933

**Published:** 2021-03-31

**Authors:** Zhenhua Song, Jin-hui Wang

**Affiliations:** ^1^Department of Pharmacology, Qingdao University School of Pharmacy, Qingdao, China; ^2^University of Chinese Academy of Sciences, Beijing, China

**Keywords:** major depressive disorder, CUMS, reward, resilience, ventral tegemental area, miRNA

## Abstract

Under chronic stress, the appearance of depression-like behaviors may be related to the decline of the brain's reward circuit function which caused by long-term lack of reward. However, the effect of reward treatment on depressive-like behaviors induced by chronic stress and its molecular mechanism in the brain remain poorly understood. Here, accompanying with companion was used to imitate a reward to study the effect of reward on depression-like behaviors induced by chronic unpredicted mild stress (CUMS), and high-throughput sequencing was used to analyze the miRNA and mRNA profiles in ventral tegmental area (VTA) harvested from depression-like and resilient behaviors mice. We observed that CUMS-induced depression-like behaviors were ameliorated by accompanying with companion in mice, and 202 differentially expressed genes (DEGs) were found to be associated with depression-like behaviors, 27 DEGs associated with resilience, 159 DEGs associated with accompanying with companion. Importantly, we also obtained 228 differentially expressed miRNAs that associated with accompanying with companion. Furthermore, the miRNA-mRNA network associated with companion was established in ventral tegmental area, based on the miRNA and mRNA profiles. Altogether, our results uncover a new way to ameliorate depression-like behavior, as well as many potential drug targets to prevent or treat depression.

## Introduction

Depression is a well-known disabling mental illness characterized by low mood, lack of interest, and lack of pleasure, accompanied by corresponding changes in thinking and behavior (1–4). The World Health Organization (WHO) reported that MDD will be the leading cause of disability and have the highest burden of disease by the year 2030 (5). Despite the societal impact of MDD, little is known about the specific etiology of the disorder. Studies of major depressive disorder have shown that lack of reward can lead the brain's reward circuit to be not fully activated or actively used, thereby reducing the function of the brain's reward circuit, including ventral tegmental area (VTA), nucleus accumbens (NAc) and Prefrontal cortex (PFC), which can lead to major depressive disorder (6–8).

The reward circuit is an integral part of brain's limbic system, which not only promotes learning, stimulating and avoiding behaviors, but also regulates cognition and motivation (9, 10). Recent studies have also demonstrated that disrupted topological organization within the reward circuit is significantly associated with cognitive deficits and depression severity in MDD patients (9, 11, 12). Thus, researchers began to consider whether rewards can be used as a positive emotional stimulus to have an effect on depression, many studies have begun to study the effect of reward intervention on depressive symptoms. Music interventions can improve the quality of sleep, quality of life, and lack of pleasure in patients (13–15). What is more, encouraging patients to participate in reward activities during treatment was found to be effective in reducing MDD symptoms (16), and positive social feedback can change mood levels and reward the value of neutral faces (17). Numerous studies confirm that few social relations and low social support, in particular low perceived emotional support, are risk factors for depression (18, 19), and poor recovery from depressive disorder has been shown to be related to low perceived social support and loneliness (20). Therefore, it will be very meaningful to test whether social relationship interventions help to better prevent depression, and the molecular changes in the reward circuit may provide better theoretical support for the application of reward intervention in the treatment of depression.

In this study, accompanying with companion was performed as a reward intervention, depression-like behavior mice, or resilient mice were induced by chronic unpredictable mild stress (CUMS), the miRNA, and mRNA profiles of VTA were analyzed by high-throughput sequencing to reveal the molecular changes. This work provides a solid basis for the understanding of the effect of reward treatments on the CUMS-induced depression-like behavior, and for the identification of reward-related genes, which may be used as an effective target for the prevention and treatment of MDD.

## Materials and Methods

### Mice

Three-week-old C57BL/6J mice were purchased from Beijing Vital River Laboratory Animal Technology Co., Ltd, and were maintained under controlled environmental conditions with free access to food and water, and illumination was provided between 07:00 and 19:00. The ambient temperature and relative humidity were maintained at 22 ± 2°C and 55 ± 5%, respectively. The experimental protocols were approved by the Animal Use and Care Committee of Qingdao University.

### Procedures for Chronic Unpredicted Mild Stress and Accompanied by Confidant Administration

**Figure 1:**
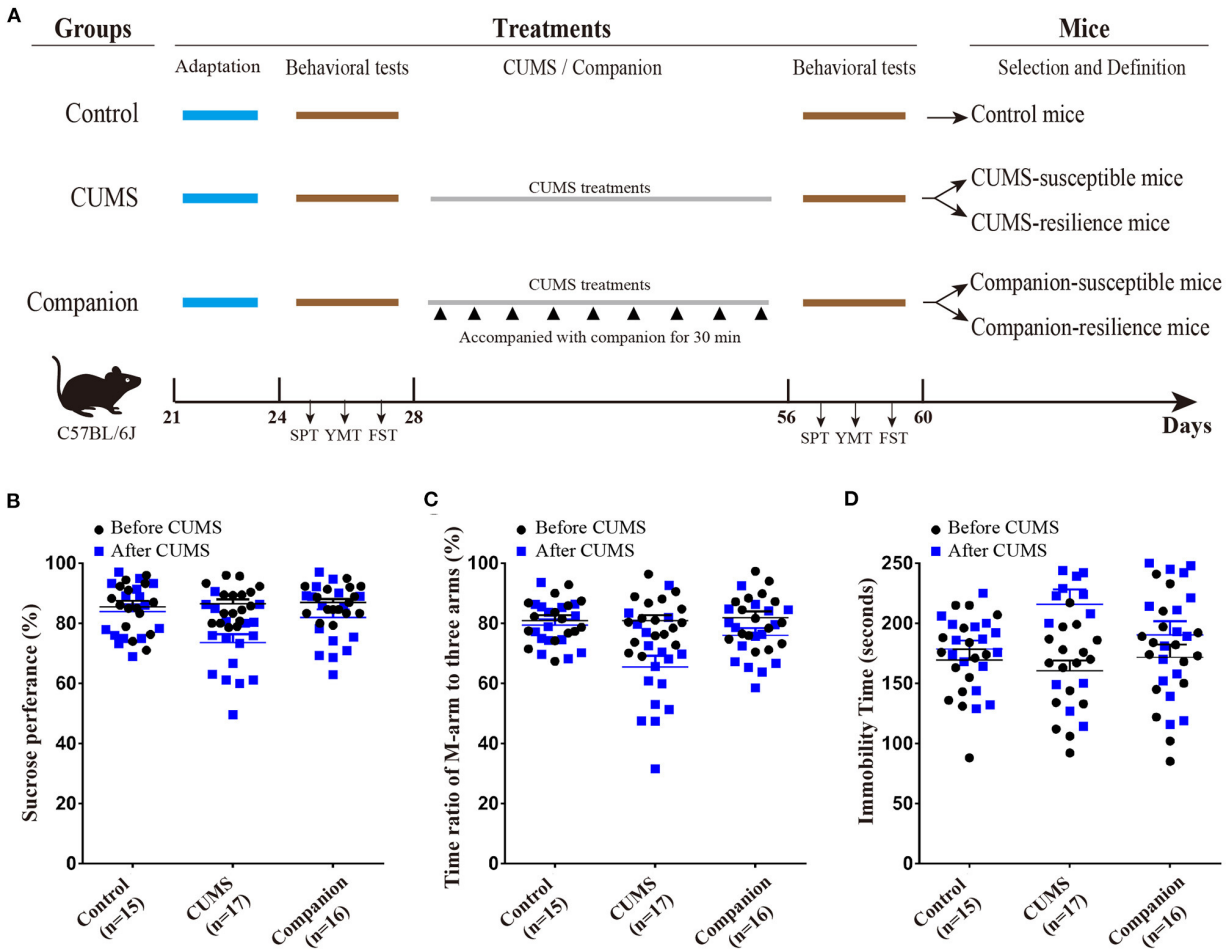
CUMS-induced depression-like behaviors were ameliorated by accompanying with companion. **(A)** Procedure for generating CUMS-susceptible, CUMS-resilience, Companion-susceptible, and Companion-resilience mice, including adaptation, behavioral tests, CUMS, CUMS with companion treatments, and definition. **(B)** Sucrose preference test values (%) before (Black) and after (blue) treatments in Control (*n* = 15), CUMS (*n* = 17), and Companion groups (*n* = 16). **(C)** Time ratios of M-arm to three arms before (Black) and after (blue) treatments in Control (*n* = 15), CUMS (*n* = 17), and Companion groups (*n* = 16). **(D)** Immobile time of staying in the water cylinder before (Black) and after (blue) treatments in Control (*n* = 15), CUMS (*n* = 17), and Companion groups (*n* = 16). Two-way ANOVA was used for the comparisons among control, CUMS, and Companion groups.

After the juvenile mice were purchased, they were acclimated for a week, in which body weight, sucrose preference (SPT), Y-maze tests (YMTs), and forced swim test (FST) were measured to have self-control data. According to the behavioral test data, juvenile male mice were randomly divided into three groups: Control group (without CUMS), CUMS group (treated by CUMS), and Companion group (treated by CUMS and accompanied with companion) (Figure 1A). The CUMS procedure consisted of a variety of unpredictable mild stressors including empty cage, tilted cage, white noise, restraint space, damp sawdust cage, strobe light, social isolation, and circadian disturbance, and were performed as previously described (21, 22). Briefly, juvenile mice in CUMS and Companion groups were maintained for 4 weeks under CUMS environment, and juvenile mice in the Companion group were accompanied with female confidant from the same litter for 30 min once per 3 days, when they were in the interval among treatments by stress (Figure 1A). Behavioral tests are performed before and after CUMS treatment, respectively.

### Selecting and Definition of MDD and Resilience Mice

After CUMS or Companion treatments, behavioral tests were conducted to determine whether the mice developed anhedonia and lack of motivation. SPT, YMT, and FST were used to evaluate anhedonia (23), loss of interest to their partners and self-esteem (24–27), respectively. As previously described (28–30), mice with significant changes in all three tests (values in the SPT and the YMT decreased above 20% of its self-control values as well as the immobile time of the FST increased 15% above its self-control values) were defined as CUMS-susceptible mice in CUMS group, Companion-susceptible mice in Companion group. Mice with <5% changes in all three tests were named as CUMS-resilience mice in CUMS group, Companion-resilience mice in Companion group.

### RNA Purification From the Medial Prefrontal Cortex

CUMS-susceptible, CUMS-resilience, Companion-susceptible, Companion-resilience, and control mice were anesthetized by using Isoflurane, perfused by normal saline in intra-heart and decapitated. The VTA (lateral VTA) was separated on ice-cold glass slide. Total RNAs from VTA were isolated in TRIzol Reagent (Life Technology, Carlsbad, CA, USA) as previously described (30), and RNA samples were delivered to Beijing Genomics Institute (BGI) in China for high-throughput sequencing analysis under dry ice conditions.

### Library Preparation and mRNA-Sequencing

mRNAs were purified with Oligo (dT)-attached magnetic beads, and sequentially fragmented into small pieces. Then, first-strand cDNA was synthesized using random hexamer-primed reverse transcription, followed by a second-strand cDNA synthesis. Obtained cDNA fragments were amplified by PCR, and products were purified by Ampure XP beads, then dissolved in EB solution. The product was validated on the Agilent Technologies 2,100 bioanalyzer for quality control. Then, undergo DSN treatment. The DSN treated library was assessed quality to ensure the high quality of the sequencing data by two methods: checked the distribution of the fragments size using the Agilent 2,100 bioanalyzer, and quantified the library using real-time quantitative PCR. The qualified library was amplified on cBot to generate the cluster on the flow cell. And the amplified flow cell was sequenced single end on the HiSeq 4,000 platform.

### Differentially Expressed Transcript Analysis and Data Analysis

The reads per kilo-base per million reads (RPKM) was used to compute the level of transcript expression. Differentially expressed transcripts of samples were determined with DEseq.2. For screening differentially expressed transcripts, the threshold used to identify DEGs were |log2 fold change| ≥0.584,936 and FDR ≤ 0.05. Subsequently, DEGs were annotated in the gene ontology (GO) database (http://www.geneontology.org/) and Kyoto Encyclopedia of Genes and Genomes (KEGG) (http://www.genome.jp/kegg~database).

### Library Preparation, miRNA-Sequencing, and Data Analysis

Library was prepared with 1 μg total RNA for each sample. Total RNA was purified by electrophoretic separation on a 15% urea denaturing polyacrylamide gel electrophoresis (GAGE) gel and small RNA regions corresponding to the 18–30 nt bands in the marker lane (14–30 ssRNA Ladder Marker, TAKARA) were excised and recovered. Then the18–30 nt small RNAs were ligated to a 5′-adaptor and a 3′-adaptor. The adapter-ligated small RNAs were subsequently transcribed into cDNA by SuperScript II Reverse Transcriptase (Invitrogen, USA) and then several rounds of PCR amplification with PCR Primer Cocktail and PCR Mix were performed to enrich the cDNA fragments. The PCR products were selected by agarose gel electrophoresis with target fragments 100–120bp, and then purified by QIAquick Gel Extraction Kit (QIAGEN, Valencia, CA). The library was quality and quantitated in two methods: check the distribution of the fragments size using the Agilent 2,100 bioanalyzer, and quantify the library using real-time quantitative PCR (TaqMan Probe). The final ligation PCR products were sequenced using the BGISEQ-500 platform.

Subsequently, contaminated reads, including adapter dimers, junk, low complexity, common RNA families (rRNA, tRNA, snRNA, and snoRNA), were disregarded to obtain clean reads. Cleaned tags were annotated with miRBase 21.0 to identify known miRNAs. miRNA expression levels were calculated with TPM values (31). DESeq software algorithm based on negative binomial distribution and biology duplicate samples was used to compare the known or novel miRNA expression among different groups. The threshold used to identify the different expression of miRNAs was fold-change larger than 1.5 and *P*-value <0.05. Three prediction approaches (RNAhybrid, Targetscan, and miRanda) were used to identify the miRNA binding sites.

### Integrated miRNA/mRNA Network Analysis

A series of bioinformatics analyses found that there is a correlationship between miRNAs and their target mRNAs. miRNAs were usually negatively correlated with their targeted mRNAs in theory, despite a few exceptions (32). To identify potential miRNA-regulated target genes, the datasets of differentially expressed miRNAs and transcripts were integrated according to the following criteria: (1) In our analysis, miRNAs and mRNAs should undergo reverse changes simultaneously; (2) The correlationship between miRNAs and their target mRNAs should be predicted by the software of RNAhybrid, Targetscan, or miRanda. Cytoscape software (San Diego, CA USA) was used to visualize the interactive network of differentially expressed miRNAs and concurrently expressed target mRNAs.

### Statistical Analyses

The data of behavioral tests were presented as mean ± SEM. Two-way ANOVA was used to make the statistic comparison among control, CUMS and Companion groups before and after treatment. Paired *t*-test was used for statistics of before vs. after values within groups. The unpaired Student *t*-test was used to make the statistic comparison between control and CUMS-susceptible, control and CUMS-resilience, control and Companion-susceptible, control and Companion-resilience, CUMS-resilience and Companion-resilience, and so on. *P* < 0.05 is considered statistically significant.

## Results

### CUMS-Induced Depression-Like Behaviors Were Ameliorated by Accompanying With Companion in Juvenile Mice

To investigate the effect of reward on CUMS-induced depression-like behaviors, CUMS model (33), as a classic model, was used to product mice with depression-like behaviors, and accompanying with companion was used to imitate a reward treatment. As shown in Figure 1A, juvenile male mice were divided into three groups, such as, Control, CUMS, and Companion group. Mice in CUMS and Companion group were treated with CUMS for 4 weeks, among which mice in Companion group were accompanied with companion once per 3 days. Then, sucrose preference test (SPT), Y-maze test (YMT), and forced swimming test (FST) were used to assess the depression-like behaviors or resilient behaviors, only mice changed significantly in all three tests could they be defined as susceptible mice, and mice did not change significantly in all three tests could they be defined as resilient mice (Figure 1A). As shown in Figures 1B–D, all three behavioral parameters of the CUMS and Companion groups varied so much after 4 weeks treatments. In CUMS group mice, the SPT values (86.53 ± 1.432 vs. 73.58 ±2.799, *p* < 0.001, *n* = 17) (Figure 1B and Supplementary Table 1) and the ratios of stay time in M-arm to stay time in total arms (80.87 ± 1.847 vs. 65.43 ± 3.821, *p* < 0.001, *n* = 17) (Figure 1C and Supplementary Table 2) significantly decreased after 4 weeks treatments, and the values of FST's immobile time were 215.9 ± 12.35 after treatments and 160.9 ± 8.635 before the CUMS treatments (*p* < 0.001, *n* = 17) (Figure 1D and Supplementary Table 3). These results suggested that juvenile mice exposed to CUMS over a long period of time exhibited depressive-like behaviors. Furthermore, it is noteworthy that pairwise comparisons among the three groups after CUMS showed significant differences in SPT, YMT, and FST, respectively (Figures 1B–D, Supplementary Tables 1–3). Excitingly, we found that the SPT and YMT values significantly increased in Companion group compared with CUMS group, and the values of FST's immobile time significantly decreased in Companion group compared with CUMS group (Figures 1B–D, Supplementary Tables 1–3). In addition, the percentage of susceptible and resilient mice in CUMS group were about 29.41% (5/17) and 17.65% (3/17), respectively, while these in Companion group were about 18.75% (3/16) and 31.25% (5/16), respectively. These results suggested that CUMS-induced depression-like behaviors were ameliorated by accompanying with companion in juvenile mice.

### mRNA Expression Profiles of VTA Were Altered by Accompanying With Companion in CUMS-Induced Susceptible and Resilience Mice

**Figure 2:**
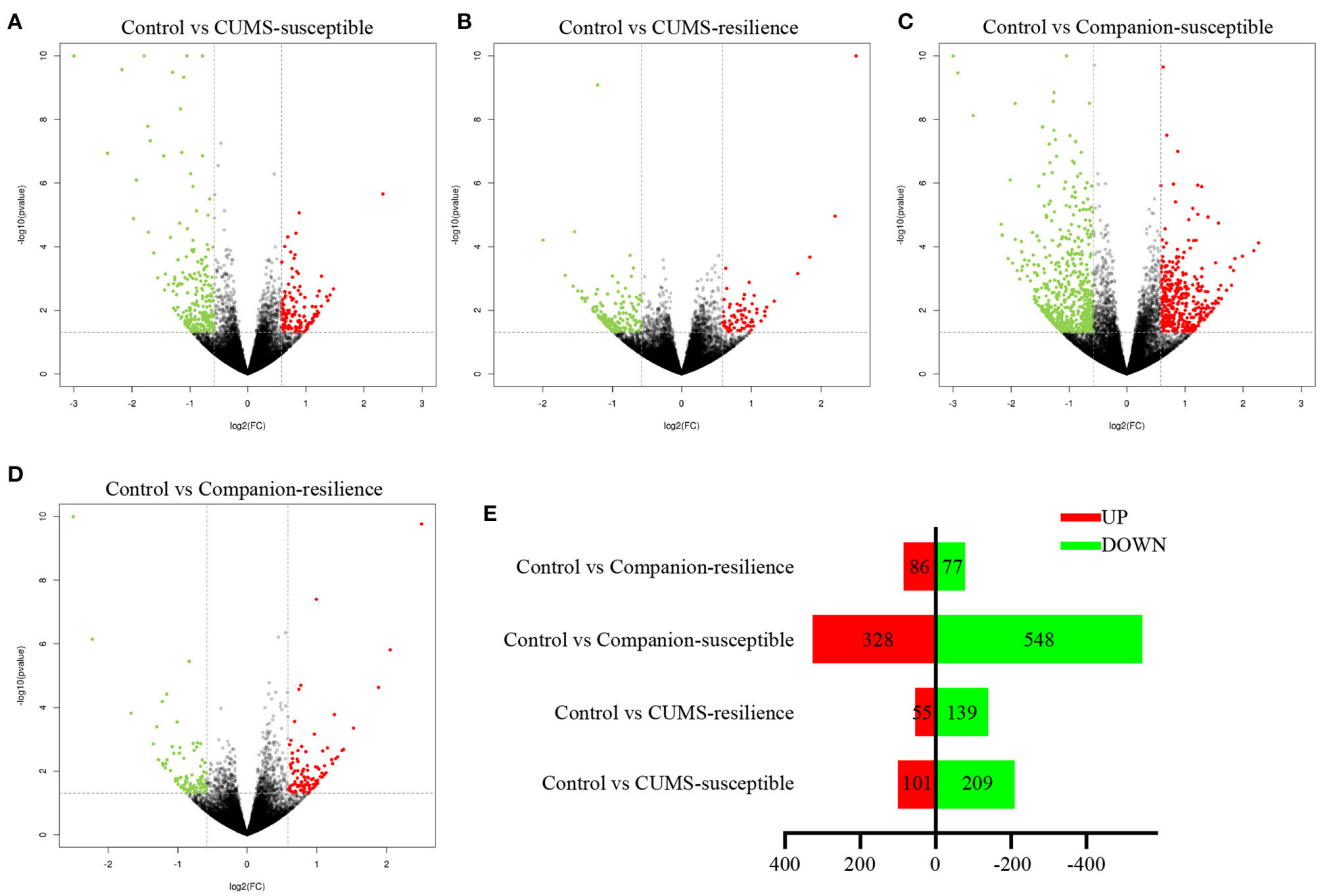
mRNA expression profiles of VTA in Control, CUMS-susceptible, CUMS-resilience, Companion-susceptible, and Companion-resilience mice. **(A–D)** The MA plot displays the DEGs distribution between Control mice and CUMS-susceptible mice, CUMS-resilience mice, Companion-susceptible mice, Companion-resilience mice, respectively. **(E)** The column chart shows the number of statistical DEGs.

In order to understand the molecular mechanism underlying accompanying with companion to ameliorate CUMS-induced depression-like behaviors, we studied the mRNA profifiles in VTA harvested from Control, CUMS-susceptible, Companion-susceptible, CUMS-resilience, and Companion-resilience by high-throughput sequencing, and screened differentially expressed mRNAs. As shown in Figures 2A–E and Supplementary Table 4, compared with the Control mice, there were 310 differentially expressed mRNAs in CUMS-susceptible mice, of which 101 mRNAs up-regulated and 109 mRNAs down-regulated. In Companion-susceptible mice, 876 mRNAs were differentially expressed compared to Control mice, of which 548 mRNAs down-regulated and 328 mRNAs up-regulated. In Control vs. CUMS-resilience mice, 194 differentially expressed mRNAs were obtained, of which 55 mRNAs upregulated and 139 mRNAs downregulated. In addition, compared to Control mice, 163 mRNAs were differentially expressed in Companion-resilience mice, of which 86 mRNAs upregulated and 77 mRNAs downregulated. These results suggested that whether in CUMS-susceptible mice or in CUMS-resilience mice, mRNA expression profiles of VTA were altered by accompanying with companion.

### Identification of the Molecular Changed in VTA Under Depression-Like Behaviors

**Figure 3:**
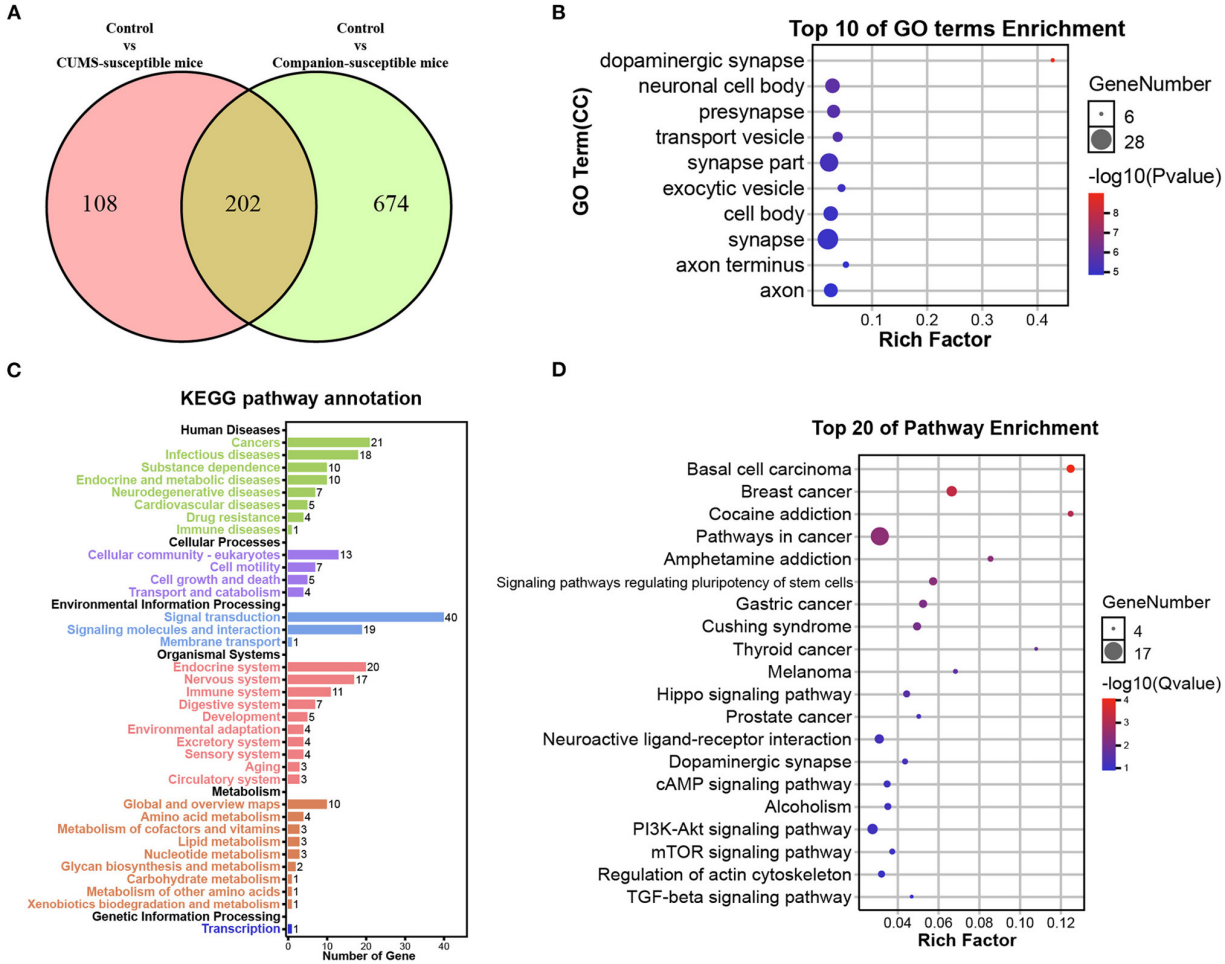
Functional analysis of DEGs associated with depression-like behaviors in VTA. **(A)** Screening of DEGs associated with depression-like behaviors in Control vs. CUMS-susceptible mice and Control vs. Companion-susceptible mice through Venn diagram. **(B)** The histogram demonstrates common DEGs GO enrichment results, CC (cellular component). **(C)** KEGG pathway annotation for common DEGs. **(D)** The histogram demonstrates common DEGs KEGG pathway enrichment results, top 20 of pathway enrichment were given.

Susceptible mice were obtained in both CUMS treated group and Companion group, respectively, and their behavioral characteristics were consistent. Thus, it is possible that certain genes are co-present in CUMS-susceptible and Companion-susceptible mice to be associated with depression-like behaviors. To test this, DEGs in Control vs. CUMS- susceptible mice and Control vs. Companion-susceptible mice were analyzed and presented using a Venn diagram (Figure 3A). The results showed that there were 108 DEGs particularly involved in Control vs. CUMS-susceptible, 674 DEGs particularly in Control vs. Companion-susceptible, and 202 common DEGs in both two comparisons (Figure 3A and Supplementary Table 5). Interestingly, regardless of whether the expression increased or decreased, each common gene has the consistent expression pattern in CUMS-susceptible and Companion-susceptible mice, compared to Control mice.

GO and Kyoto Encyclopedia of Genes and Genomes (KEGG) pathway enrichment analysis were performed to obtain better insights into the molecular function of these common DEGs associated with depression-like behaviors in VTA. DEGs were found to be enriched in GO terms associated with dopaminergic synapse, neuronal cell body, presynapse, transport vesicle, synapse part, exocytic vesicle, cell body, synapse, axon terminus, and axon (Figure 3B). KEGG pathway annotation results showed that DEGs mainly participate in signal transduction and are enriched in endocrine and nervous system (Figure 3C). Moreover, DEGs were found to be mainly enriched in KEGG pathways associated with cocaine addition, amphetamine addition, hippo signaling pathway, neuroactive ligand-receptor interaction, dopaminergic synapse, cAMP signaling pathway (Figure 3D).

### Identification of the Molecular Changed in VTA Under Resilient Behaviors

**Figure 4:**
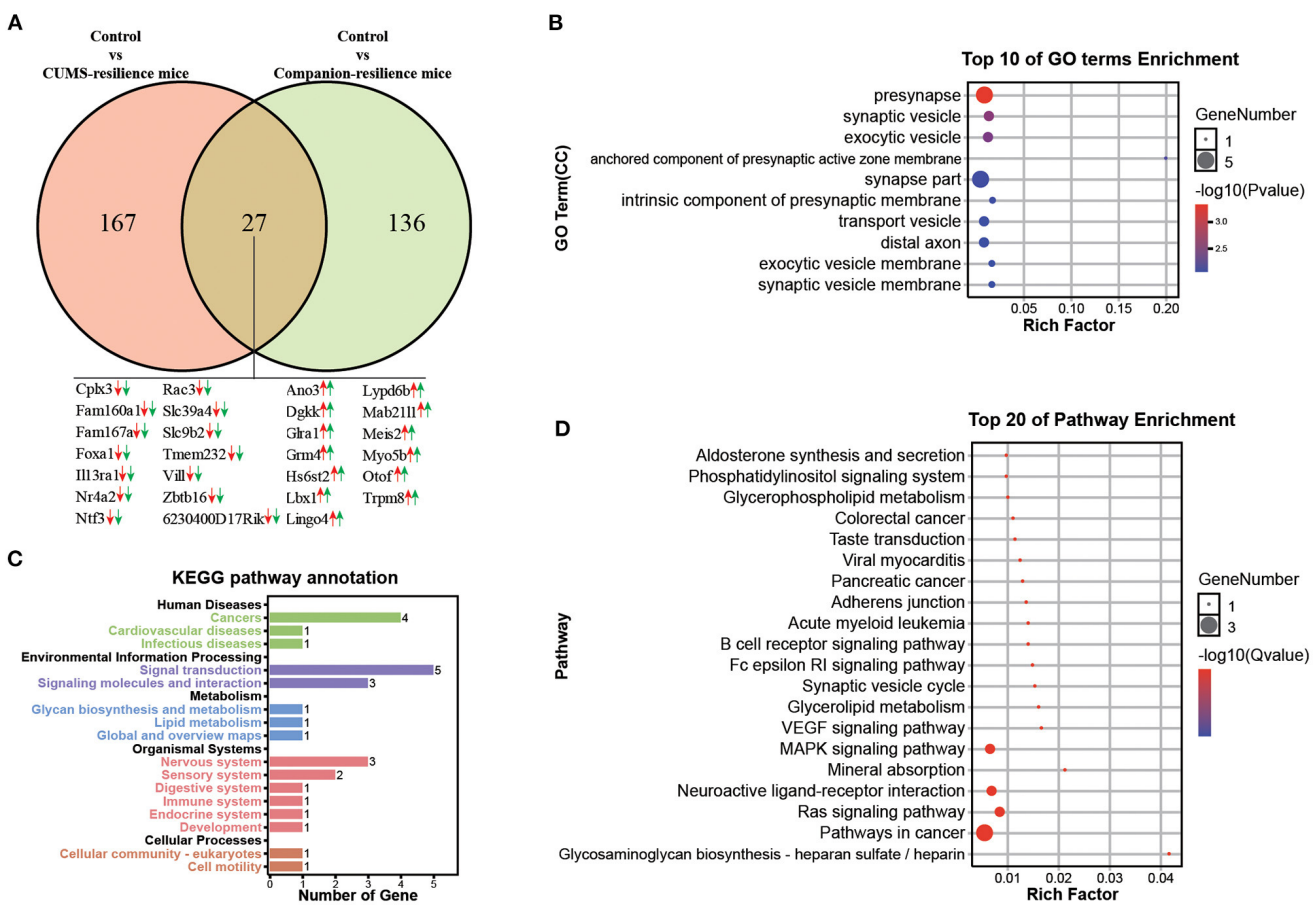
Functional analysis of DEGs associated with resilience in VTA. **(A)** Screening of DEGs associated with resilience in Control vs. CUMS-resilience mice and Control vs. Companion-resilience mice through Venn diagram. **(B)** The histogram demonstrates common DEGs GO enrichment results, CC (cellular component). **(C)** KEGG pathway annotation for common DEGs. **(D)** The histogram demonstrates common DEGs KEGG pathway enrichment results, top 20 of pathway enrichment were given.

Similar to the susceptible mice, mice with resilient behaviors were selected from both CUMS group and Companion group according to the consistent behaviors. Therefore, genes associated with resistance may be present in both CUMS-resilience mice and Companion-resilience mice. To test this, DEGs in Control vs. CUMS-resilience and Control vs. Companion-resilience were identified and presented in a Venn diagram (Figure 4A). As shown in Figure 4A, there were 167 DEGs particularly involved in CUMS-resilience mice compared to Control mice, 136 unique DEGs in Companion-resilience compared to Control mice (Figure 4A and Supplementary Table 6). Surprisingly, compared with susceptible mice, both resilience mice appeared to share less molecular changes, only 27 common DEGs were identified in both two comparisons. However, each common gene have the consistent expression pattern in CUMS-susceptible and Companion-susceptible mice compared to control mice.

To gain in-depth insights into the molecular function of 27 common DEGs, we performed GO and KEGG enrichment analysis. As shown in Figure 4B, DEGs were found to be enriched in GO terms associated with presynapse, synapse vesicle, exocytic vesicle, anchored component of presynaptic active zone membrane, synapse part, intrinsic component of presynaptic membrane, transport vesicle, exocytic vesicle membrane, synapse vesicle membrane. Moreover, KEGG pathway annotation results showed that DEGs mainly participate in signal transduction and are enriched in nervous system (Figure 4C). In addition, DEGs were found to be mainly enriched in KEGG pathways associated with pathway in cancer, neuroactive ligand-receptor interaction, MAPK signaling pathway, Ras signaling pathway, and synapse vesicle cycle (Figure 4D).

### The Changes of mRNA Expression in the VTA Between CUMS-Resilience and Companion-Resilience

**Figure 5:**
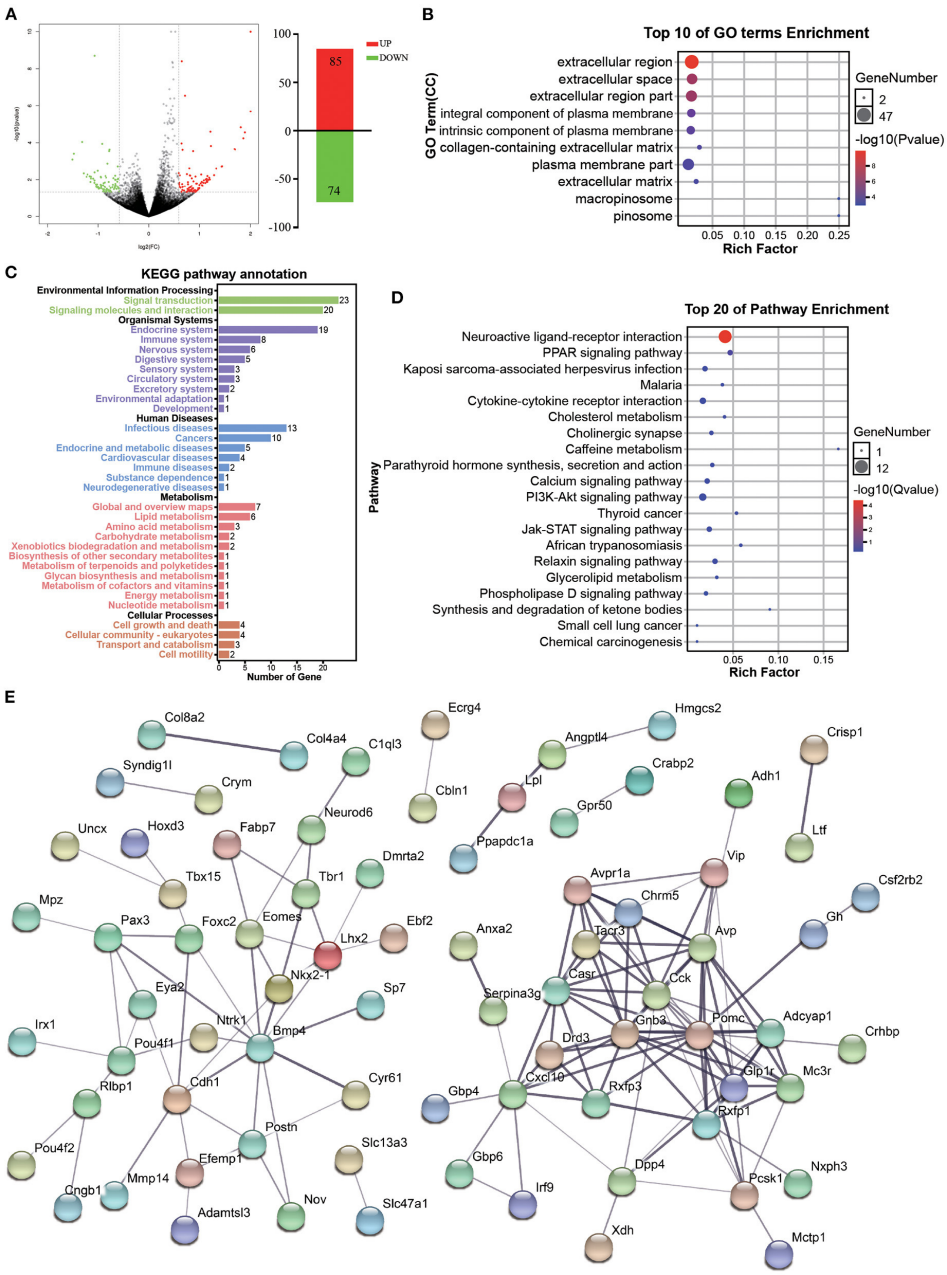
Functional analysis of DEGs associated with accompanying with companion in VTA. **(A)** The MA plot displays the DEGs distribution between CUMS-resilience mice and Companion-resilience mice, the column chart on the right is the number of statistical DEGs. **(B)** The histogram demonstrates DEGs GO enrichment results, CC (cellular component). **(C)** KEGG pathway annotation for DEGs. **(D)** The histogram demonstrates DEGs KEGG pathway enrichment results, top 20 of pathway enrichment were given. **(E)** PPI network of the DEGs visualized by Cytoscape.

In the population, although some people have experienced long-term chronic stress, they do not show depression-like behaviors, i.e., resilience (34). In our previous research, we also found the existence of resilience mice after CUMS treatments (28–30), and accompanying with companion increased the appearance of resilience (21). In order to understand the molecular changes caused by accompanying with companion, DEGs associated with accompanying with companion were screened in VTA between CUMS-resilience and Companion-resilience. Compared with the CUMS-resilience mice, 159 mRNAs were differentially expressed in Companion-resilience, of which 85 mRNAs were up-regulated and 74 mRNAs were down-regulated (Figure 5A and Supplementary Table 4).

Subsequent GO term results showed that DEGs associated with accompanying with companion were enriched in extracellular region, extracellular space, extracellular region part, integral component of plasma membrane, intrinsic component of plasma membrane, plasma membrane part, and extracellular matrix (Figure 5B). Furthermore, KEGG pathway annotation results showed that DEGs mainly participate in signal transduction and signaling molecules and interaction, and are enriched in endocrine, immune and nervous system (Figure 5C). DEGs were found to be mainly enriched in KEGG pathways associated with PPAR signaling pathway, neuroactive ligand-receptor interaction, cytokine-cytokine receptor interaction, cholinergic synapse, calcium signaling pathway, and PI3K-Akt signaling pathway (Figure 5D). Additionally, by using the STRING database to predict the protein-protein interactions, most DEGs formed a cluster including *Cck, Gnb3, Pomc, Glp1r, Drd3, Casr, Tacr3, Rxfp1, Cxcl10, Avpr1a, Chrm5, Avp, Adcyap1, Mc3r*, and *Pcsk1* (Figure 5E).

### Accompanying With Companion Altered miRNA Expression in VTA in CUMS-Induced Resilience Mice

**Figure 6:**
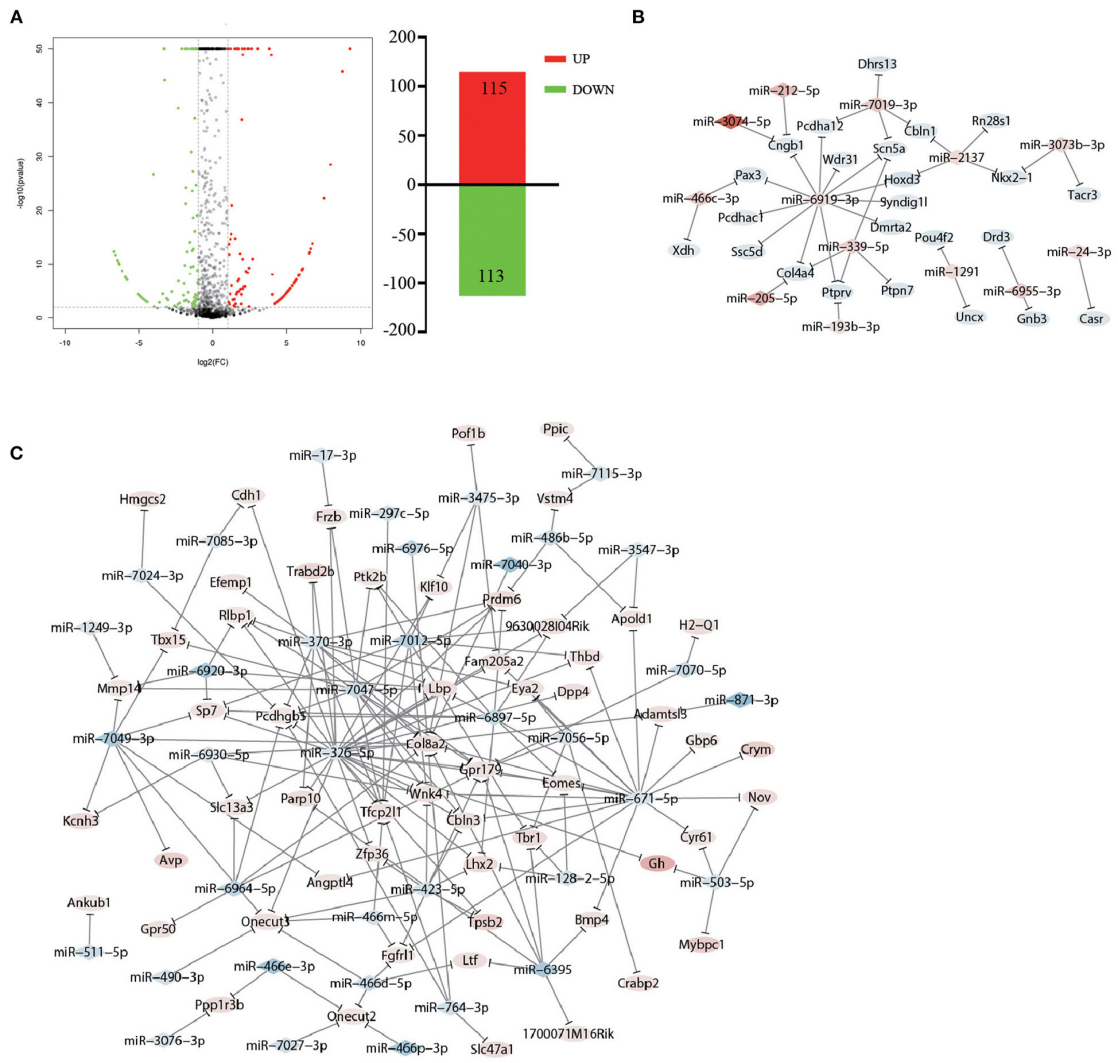
miRNA expression in resilience mice was altered after accompanying with companion. **(A)** The MA plot displays the differently expression miRNAs distribution between CUMS-resilience mice and Companion-resilience mice, the column chart on the right is the number of statistical differently expression miRNAs. **(B,C)** miRNA-mRNA network in CUMS-resilience mice vs. Companion-resilience mice. The regulatory relationship was established between 50 miRNAs in miRNA profile and 82 DEGs in mRNA profile by the prediction from RNAhybrid, Targetscan, and miRanda databases. Diamonds symbols indicate miRNAs, ovals indicate mRNAs. The shades of red present the strength of the up-regulation of miRNA and mRNA. The shades of blue present the strength of the down-regulation of miRNA and mRNA.

Studies have revealed that miRNAs play multiple roles in psychiatric disorders (35), and manipulating the levels of specific miRNAs in the brain can change behavior (36). Thus, we propose the hypothesis that accompanying with companion may induce miRNA changes in VTA, which in turn cause changes in gene expression to ameliorate CUMS-induced depression-like behaviors. To test whether the accompanying with companion alters the miRNA changes in VTA, we performed differential expression analysis of miRNA in CUMS-resilience mice and Companion-resilience mice by high-throughput sequencing. Compared with the CUMS-resilience mice, a total of 115 differentially expressed miRNAs were up-regulated and 113 differentially expressed miRNAs were down-regulated in the Companion-resilience mice (Figure 6A and Supplementary Table 7). Furthermore, to establish the miRNA-mRNA regulation network, we used the RNAhybrid, Targetscan, and miRanda databases to predict the miRNAs target genes, after which target genes were compared with DEGs associated with accompanying with companion in VTA for screening out mRNAs regulated by differentially expressed miRNAs. As shown in Figures 6B,C and Supplementary Table 8, 50 miRNAs established regulatory relationships with 82 mRNAs in CUMS resilience vs. Companion-resilience mice.

## Discussion

Numerous researches have shown that major depressive disorder was caused by the interaction of reward, stress regulation and mentalizing (37). The individual's emotion regulation ability can be effectively promoted by reward-induced motivation (38), while lack of reward can lead the brain's reward circuit to be not fully activated or actively used, thereby reducing the function of the brain's reward circuit (39). While encouraging patients to engage in rewarding activities during treatment can be effective in reducing the symptoms of major depressive disorder (16). In this study, CUMS treatments induced depression-like behaviors in juvenile mice. Accompanying with companion was performed as a natural reward treatment, and ameliorated the CUMS-induced depression-like behaviors in juvenile mice (Figures 1B–D). Subsequently, to gain insight into molecular functions, high-throughput sequencing was performed on mRNA and miRNA in VTA from CUMS-susceptible, Companion-susceptible, CUMS-resilience and Companion- resilience mice, and many genes related to depression-like behaviors, resilience and accompanying with companion were obtained.

Affective disorders are thought to be caused by complex interactions between genetic vulnerability and adverse environmental events (40). If a single or a combination of multiple pathogenic factors reach a critical size or occur simultaneously, these factors may cause transient or even continuous dysfunction changes in several brain regions and systems, which will lead to the occurrence of depression (41). Thus, depression is a multifactorial and multigenetic disease, and its molecular and biochemical mechanisms is complex. In this study, we obtained depression-like behavior mice from CUMS and Companion groups based on consistent behavioral performance. However, they still showed large differences in gene expression in VTA at the molecular level. This implies that the molecular mechanisms of depression caused by different environmental factors may be different. Even so, we still found 202 differentially expressed genes with similar expression patterns in the comparisons between Control vs. CUMS- susceptible and Control vs. Companion- susceptible mice, and defined these genes as DEGs associated with depression-like behaviors. Subsequent GO and KEGG pathway analysis found that these genes mainly enriched in synapse, neuronal cell body, axon and transport vesicle, and mainly participated in signal transduction and signaling molecules and interaction. This indicates that there are still larger molecular changes underlying the symptoms of depression-like behavior. Therefore, more research is needed to understand how important these genes are for the prevention and treatment of depression.

Different individuals respond to stress differently, some suffer from psychological disorders related to stress, some have mild to moderate psychological symptoms that quickly disappear, and some do not develop new psychological symptoms, i.e., resilience (34). In this study, we also obtained resilience mice from CUMS and Companion group, respectively. Although resilience derived from two groups were behaviorally consistent, there were only 27 common DEGs in the comparisons between Control vs. CUMS-resilience and Control vs. Companion-resilience, which was in sharp contrast to 202 DEGs associated with depression-like behaviors. Therefore, resilience to stress is a complex multidimensional construct with complex molecular mechanisms.

Many studies have shown that aspects of emotional support were significantly associated with depression outcome, low perceived social support and loneliness were related to depression course and recovery, and positive family support can help recover from a major depressive episode (18–20). Therefore, it is particularly important to detect the contribution of a partner or close friend in prevention of depression-like behavior and its molecular mechanisms. In this study, mice treated by CUMS and the companion used for accompanying were from the same litter, which is a good way to simulate a partner or close friend. As we expected, we found that there was a good effect in ameliorating depression-like behaviors after accompanying with companion, and this treatment also triggered a series of molecular changes. Associated with accompanying with companion, 159 mRNAs and 228 miRNAs were identified, from which 50 miRNAs and 82 mRNAs were used to establish the miRNA/mRNA network. Thus, our future work will focus on the regulation of miRNA/mRNA to reveal whether molecular changes has the same effect as companion in ameliorating depression-like behaviors.

In chronic unpredicted mild stress, the social isolation and the worst living conditions are added to mice. To relieve stressful situations by providing good situations for mice to improve their depressive behaviors, rewards are given to mice experiencing CUMS by providing food and social communication, especially communication with their patterners, which are expected to be against stressful situations. As there is no quantified protocol for the rewards, we have used the companion communication for stressed mice to meet their patterners 30 min every 3 days. By this design and protocol, we expect to improve stress-induced depression and to detect molecular profiles. This study is expected to reveal potential protocols as well as mechanisms underlying the improvement of stress-induced behaviors.

In conclusion, we found that CUMS-induced depression-like behaviors were ameliorated by accompanying with companion, with significant changes in mRNA and miRNA in VTA. These molecules may be applied to the prevention and treatment of depressive disorder, such as, potential nucleic acid drugs and potential drug targets for depression. In addition, this study not only provided a potential method to prevent depression, but also provided theoretical support for reward intervention in the treatment of depression.

## Data Availability Statement

The data presented in the study are deposited in the (SRA database) repository, accession number (PRJNA695872).

## Ethics Statement

The animal study was reviewed and approved by Animal Use and Care Committee of Qingdao University.

## Author Contributions

ZS performed the experiments and data analyses. ZS and J-hW initiated the project, designed the experiments, and wrote the paper. All authors read and approved the final manuscript.

## Conflict of Interest

The authors declare that the research was conducted in the absence of any commercial or financial relationships that could be construed as a potential conflict of interest.
