# Supplementary material for: miRNA and mRNA Profiles in Ventral Tegmental Area From Juvenile Mice With Companion Communication of Improving CUMS-Induced Depression-Like Behaviors
Source: Front Psychiatry. 2021 Mar 31;12:634933. doi: 10.3389/fpsyt.2021.634933 (PMC8044319; doi:10.3389/fpsyt.2021.634933)
Supplement: Supplementary file 1 [file Table_1.DOCX]

**Table S1. Double-tailed analysis of Sucrose preference test (SPT) in Figure 1.**

| **Period of comparison** | **Multiple comparisons** | **Mean Difference.** | **Significant?** | **Summary** | **Adjusted P-value** |
| --- | --- | --- | --- | --- | --- |
| **Before CUMS** | Control vs CUMS | -1.07 | No | ns | 0.9798 |
|  | Control vs Companion | -1.444 | No | ns | 0.9546 |
|  | CUMS vs Companion | -0.3746 | No | ns | 0.9990 |
| **After CUMS** | Control vs CUMS | 10.32 | Yes | ** | 0.0033 |
|  | Control vs Companion | 1.923 | No | ns | 0.9012 |
|  | CUMS vs Companion | -8.395 | Yes | * | 0.0193 |
| **Before CUMS-**  **After CUMS** | Control | 1.564 | No | ns | 0.9921 |
|  | CUMS | 12.95 | Yes | *** | <0.0001 |
|  | Companion | 4.931 | No | ns | 0.0002 |

Note. Three asterisks show p < 0.001, two asterisks show p < 0.01, one asterisk show p < 0.05, in which two-way ANOVA was used for the comparisons among control group, CUMS group, and CUMS-Confidant group, and paired t-test was used for analysis of before versus after values within groups.

**Table S2. Double-tailed analysis of Y-maze test (YMT) in Figure 1.**

| **Period of comparison** | **Multiple comparisons** | **Mean Difference.** | **Significant?** | **Summary** | **Adjusted P-value** |
| --- | --- | --- | --- | --- | --- |
| **Before CUMS** | Control vs CUMS | 0.02239 | No | ns | >0.9999 |
|  | Control vs Companion | -1.022 | No | ns | 0.9884 |
|  | CUMS vs Companion | -1.045 | No | ns | 0.9865 |
| **After CUMS** | Control vs CUMS | 13.98 | Yes | *** | 0.0004 |
|  | Control vs Companion | 3.382 | No | ns | 0.7161 |
|  | CUMS vs Companion | -10.6 | Yes | ** | 0.0081 |
| **Before CUMS-**  **After CUMS** | Control | 1.488 | No | ns | 0.9674 |
|  | CUMS | 15.44 | Yes | **** | <0.0001 |
|  | Companion | 5.892 | No | ns | 0.2579 |

Note. Four asterisks show p < 0.0001, Three asterisks show p < 0.001, two asterisks show p < 0.01, in which two-way ANOVA was used for the comparisons among control group, CUMS group, and CUMS-Confidant group, and paired t-test was used for analysis of before versus after values within groups.

**Table S3.** **Double-tailed analysis of Forced swimming test (FST) in Figure 1.**

| **Period of comparison** | **Multiple comparisons** | **Mean Difference.** | **Significant?** | **Summary** | **Adjusted P-value** |
| --- | --- | --- | --- | --- | --- |
| **Before CUMS** | Control vs CUMS | 8.996 | No | ns | 0.8974 |
|  | Control vs Companion | -2.221 | No | ns | 0.9982 |
|  | CUMS vs Companion | -11.22 | No | ns | 0.8131 |
| **After CUMS** | Control vs CUMS | -37.28 | Yes | * | 0.0323 |
|  | Control vs Companion | -11.9 | No | ns | 0.8002 |
|  | CUMS vs Companion | 25.38 | No | ns | 0.2088 |
| **Before CUMS-**  **After CUMS** | Control | -9.133 | No | ns | 0.9014 |
|  | CUMS | -55.41 | Yes | *** | 0.0004 |
|  | Companion | -18.81 | No | ns | 0.4723 |

Note. Three asterisks show p < 0.001, one asterisk show p < 0.05, in which two-way ANOVA was used for the comparisons among control group, CUMS group, and CUMS-Confidant group, and paired t-test was used for analysis of before versus after values within groups.
